# Error in Byline

**DOI:** 10.1001/jamanetworkopen.2023.45388

**Published:** 2023-11-10

In the Special Communication titled “Leveraging the Expertise of the CTSA Program to Increase the Impact and Efficiency of Clinical Trials,”^1^ published online October 5, 2023, there was an error in the byline. Daniel E. Ford’s middle initial was corrected. This article was corrected.^1^

## References

[1] Harris PA, Dunsmore SE, Atkinson JC, ; Trial Innovation Network. Leveraging the expertise of the CTSA program to increase the impact and efficiency of clinical trials. JAMA Netw Open. 2023;6(10):e2336470. doi:10.1001/jamanetworkopen.2023.36470 37796498 PMC10773966

